# Interference Suppression via Joint Interference Alignment and Power Allocation in Integrated Communication and Navigation Systems

**DOI:** 10.3390/s25226905

**Published:** 2025-11-12

**Authors:** Xinglong Wang, Tiantian He, Xiyuan Ren, Xiaorong Jing

**Affiliations:** 1 Institute of Telecommunication and Navigation Satellites, China Academy of Space Technology, Beijing 100094, China; wangxinglong1987@163.com; 2 Shenzhen Edadoc Technology Co., Ltd., Shenzhen 518000, China; 18483633721@163.com; 3 School of Communications and Information Engineering, Chongqing University of Posts and Telecommunications, Chongqing 400065, China; renxy@stu.cqupt.edu.cn

**Keywords:** integrated communication and navigation (ICAN), interference alignment (IA), power allocation (PA), interference suppression

## Abstract

This paper addresses the complex interference in integrated communication and navigation (ICAN) systems by proposing an interference suppression scheme that integrates interference alignment (IA) with power allocation (PA). In the proposed scheme, the IA algorithm first mitigates inter-satellite and intra-satellite beam interference by aligning interference signals into a common subspace. These signals are then eliminated through the design of beam precoding vectors and integrated user detection vectors. For intra-beam interference, we formulate a PA-based optimization problem to maximize the system sum rate. This problem is decoupled into per-beam sum rate maximization. By reformulating the user minimum rate constraints, the original problem is further transformed into a convex optimization problem. The optimal solution is derived using the Karush–Kuhn–Tucker (KKT) conditions from convex optimization theory. Simulation results demonstrate the effectiveness of the proposed interference suppression scheme.

## 1. Introduction

The integration of geostationary orbit (GEO) and non-geostationary orbit (NGEO) satellite communications has advanced rapidly, emerging as the foundation of international spatiotemporal information services. In parallel, the commercialization of fifth-generation (5G) mobile communication systems has accelerated the development of intelligent industries driven by location-based services (LBS), such as smart cities, autonomous driving, and the 5G Internet of Things. Compared with traditional industries, these emerging industries require future networks to deliver seamless global coverage, massive connectivity, ultra-reliable low-latency communication, and high-precision positioning [1]. Consequently, space–air–ground integrated communication and navigation (ICAN) networks are considered a key enabler of this vision [2]. In [3], the authors presented a comprehensive survey of ICAN technologies for Low Earth Orbit (LEO)-enabled networks and discussed their applications in various emerging scenarios, including smart city and massive IoT device access. To this end, the Third Generation Partnership Project (3GPP) had updated the New Radio (NR) protocol to incorporate satellite communication, satellite navigation, and 5G, thereby addressing the challenges of non-terrestrial networks (NTNs) [4]. At the same time, national governments and enterprises had launched initiatives such as Starlink, SpaceX, OneWeb, and Telesat [5].

Extensive research on space–air–ground ICAN networks has investigated a range of approaches. For example, ref. [6] analyzed three ICAN implementation methods for LEO-enhanced GNSS systems. Given that LEO satellites in NGEO constellations provided shorter round-trip delays, lower path loss, and reduced launch costs [7,8], Huawei proposed an integrated communication and navigation framework based on the 3GPP standard [9] to exploit the potential of ultra-dense LEO satellite networks. In [10], the integration of terrestrial and LEO satellite communication systems was investigated, including discussions on beyond 5G (B5G) air interfaces, as well as novel architectures and transmission techniques for sixth-generation (6G) networks. Based on the IEEE 802.16 WiMAX standard, ref. [11] designed the integrated communication–navigation signals using orthogonal frequency-division multiplexing (OFDM), aiming to meet the requirements of both high-speed data transmission and positioning. Building on this work, Fernández, A. et al. investigated the feasibility of applying such signals to ICAN for spacecraft formation flying, demonstrating their suitability for Mbps-level inter-satellite links [12]. In [13], navigation signals were employed as pilot signals for synchronization, while cyclic code shift keying (CCSK) signals served as communication signals. Cyclically shifted pseudorandom codes were used to enhance transmission rates. Similarly, Zhu, H. et al. leveraged existing radio signals and a signal selection algorithm to enable both communication and positioning in navigation environments [14]. More recently, ref. [15] proposed an innovative integrated communication and navigation waveform design that combines index modulation with OFDM technology. This design enhanced the system’s resilience to frequency offsets and interference. However, most existing studies have primarily focused on signal integration design in ICAN networks. In practical ICAN systems, spectrum reuse introduces severe interference challenges.

Existing studies on interference have primarily focused on multibeam satellite communication systems. In such systems, frequency reuse across spot beams maximized spectral efficiency and enabled service provision to multiple user terminals across different beam coverage areas [16]. To mitigate inter-beam interference, multicolor frequency reuse schemes were commonly employed by combining distinct frequency bands with orthogonal polarizations. For example, ref. [17] proposed a four-color reuse scheme for the forward and return links in Ka-band satellite systems. Under frequency reuse, spectrum must be allocated to spatially separated beams. To further enhance efficiency, a full frequency reuse (FFR) scheme was introduced [18], in which all beams shared the same band, although FFR causes stronger and more widespread inter-beam interference [19]. To suppress such interference, ref. [20] proposed a joint multiuser precoding and power allocation (PA) design for the forward link of fixed multibeam systems, which provided a general optimization framework under both linear and nonlinear power constraints. Ref. [21] addressed multigateway multibeam systems using channel-matrix regularized singular value block decomposition to minimize both intra- and inter-cluster interference, but the system adopted time-division multiplexing (TDM), restricting each beam to serving one user at a time. To address this limitation, ref. [22] applied multibeam multiple-input multiple-output (MIMO) technology, enabling simultaneous multiuser access in the same frequency–time domain and investigates zero-forcing (ZF) precoding for the downlink. From the perspective of energy efficiency (EE), ref. [23] studied joint beam and PA in multibeam LEO systems, while ref. [24] explored precoding design in multicast satellite systems under total power and quality-of-service (QoS) constraints. However, when large-scale user access was required, frequency reuse alone becomed insufficient. To address this limitation, ref. [25] analyzed the feasibility of power-domain non-orthogonal multiple access (NOMA) in satellite networks. For NOMA systems, ref. [26] investigated power allocation based on the max–min fairness (MMF) criterion to enhance system performance. Building on this, ref. [27] applied NOMA to satellite IoT to support massive device connectivity, while ref. [28] extended NOMA to inter-satellite uplink and downlink communications and analyzed bit error rate and throughput through simulations. Ref. [29] further optimized PA in NOMA-enabled multibeam LEO systems to maximize system throughput. Considering dynamic satellite characteristics, ref. [30] proposed a two-layer GEO/LEO network model integrating NOMA, and ref. [31] investigated joint user pairing and PA within this framework.

To meet the massive terminal access demands of emerging LBS industries, non-orthogonal multiple access (NOMA) had been introduced into ICAN systems. Although NOMA provided multiplexing gains, it also introduced intra-beam interference in addition to inter-beam interference. However, research on interference management in ICAN systems remains limited, with most existing work focusing on multibeam satellite systems. For example, ref. [32] investigated multibeam multicast NOMA (MB-MC-NOMA) satellite systems, applying precoding and NOMA techniques to mitigate inter-beam and intra-beam interference, respectively. Ref. [33] applied NOMA to large-scale MIMO LEO satellite systems, where precoding vector design suppresses inter-beam interference and power allocation maximizes the system sum rate. Similarly, ref. [34] optimized transmit covariance matrix design in large-scale MIMO LEO systems to maximize the ergodic sum rate, reformulating the problem into a precoding vector design to suppress inter-beam interference. However, these studies were limited to single-satellite scenarios. In practice, interference management across multi-satellite systems was more realistic. To this end, ref. [35] proposed zero-forcing (ZF) and regularized ZF (RZF) precoding strategies for dual-satellite systems to suppress inter-beam interference. In [36], a joint beamforming design and satellite selection optimization problem for LEO-ICAN networks was formulated. Although it directly addresses ICAN beam design and presents models and algorithms that account for inter-beam leakage and beam selection to mitigate interference, it does not consider inter-satellite or intra-beam interference. The authors in [37] analyzed the trade-off between communication and navigation performance based on the multi-beam satellite architectures. The author in [21] discussed the precoding-based suppression techniques. However, its primary focus was on single multibeam satellite systems, rather than on multi-satellite systems or ICAN systems. The work in [38] leveraged signal design to address the challenges of interference among integrated signals, rather than directly developing an interference suppression scheme. An integrated OFDM-based signal system that incorporates sidetone signals to support both communication and measurement was presented in [39]. In [40], the authors addressed the distributed multi-user detection problem under inter-satellite link (ISL) constraints, which was highly relevant to multi-user interference management in spaceborne systems. Interference alignment (IA), an effective method for interference mitigation, had been extensively studied in terrestrial wireless networks, including X channels [41], multiuser interference channels [42], heterogeneous networks [43,44], and cognitive radio networks [45]. In contrast, studies on IA in satellite communications remain scarce, with [46] providing only a preliminary investigation of subspace interference alignment for the uplink interference channel in multibeam satellite systems. In addition to IA-only and PA-only designs, hybrid IA–PA schemes have also been investigated. For example, ref. [47] proposed a framework that combines two PA methods, namely maximizing the signal-to-leakage-plus-noise ratio (Max-SLNR) at the transmitter and maximizing the signal-to-interference-plus-noise ratio (Max-SINR) at the receiver. These studies demonstrate the potential of integrating IA with PA to enhance multiuser communication performance. All these works may mention the interference issues, but they did not present a specific scheme to effectively handles inter-satellite, inter-beam and intra-beam interference together. In fact, multi-satellite ICAN systems introduce intricate interference coupling across beams and satellites that is absent in single-satellite deployments. This complexity cannot be effectively handled by conventional approaches that treat IA and PA separately. Furthermore, existing literature lacks a systematic study on a joint IA and PA design tailored for multi-satellite interference environments. These deficiencies highlight the necessity of the proposed framework in this paper, which explicitly integrates inter-satellite interference alignment with PA optimization in a unified manner.

Building on the above analysis, the rapid convergence of satellite communications, and navigation has positioned ICAN systems as a key enabler for emerging applications such as smart cities and massive IoT. However, practical ICAN deployments face severe interference challenges due to spectrum reuse, including inter-satellite, intra-satellite inter-beam, and intra-beam interference, which significantly degrade system performance. While prior studies have largely focused on signal integration or interference management in single-satellite multibeam systems, they fall short in addressing the complex interference scenarios of multi-satellite ICAN networks. Moreover, although IA has proven effective in terrestrial networks, its potential in satellite-based ICAN remains underexplored. Motivated by these gaps, this work proposes a joint IA and PA scheme that effectively suppresses multi-source interference in ICAN systems, thereby improving spectral efficiency, ensuring user quality-of-service, and enabling large-scale connectivity in interference-limited environments. The main contributions of this work are summarized as follows:

- An ICAN system model is established following the 3GPP NTN architecture, integrating navigation satellites, multibeam satellites, and terrestrial mobile communications. Based on it, an interference suppression scheme that integrates IA with PA is proposed. Unlike existing IA-only or PA-only approaches, which address only part of the interference problem, the proposed joint IA and PA scheme provides a unified framework that simultaneously mitigates inter-satellite, inter-beam, and intra-beam interference in ICAN systems. This unified design ensures the interference suppression of the inter-satellite, the inter-beam interference, and the intra-beam interference, which existing IA-only or PA-only schemes cannot achieve.
- For inter-satellite and multibeam interference in LEO systems, IA is applied to align interference into a subspace. A unified user detection vector is designed to cancel inter-satellite interference, while precoding vectors at the serving LEO satellite are optimized to suppress residual inter-beam interference.
- To mitigate intra-beam interference, the system sum-rate maximization problem with PA is decoupled into per-beam sum-rate subproblems. Each subproblem is proven convex, and the optimal solution is derived using convex optimization theory and Karush–Kuhn–Tucker (KKT) conditions.

The rest of this paper is organized as follows. Section 2 describes the ICAN system model. Section 3 presents the design and analysis of the joint IA and PA interference suppression scheme and examines its computational complexity. Section 4 reports simulation results and discussions. Finally, Section 5 concludes the paper.

## 2. System Model

### 2.1. System Configuration

**Figure 1:**
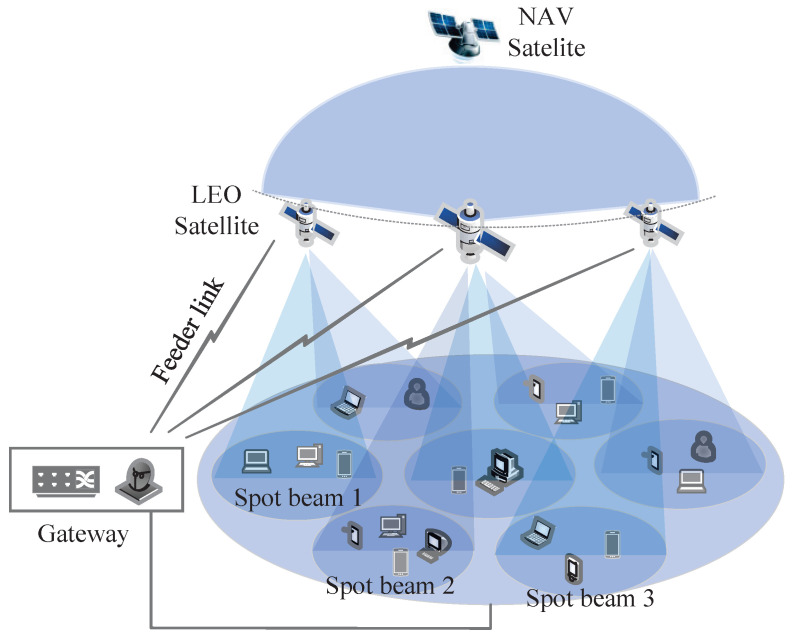
System configuration.

Consider the ICAN multiuser scenario illustrated in Figure 1, where three LEO satellites and one navigation (NAV) satellite provide coverage. The *i*th LEO satellite is denoted by $S_{i}$, with i=1,2,3. Each LEO satellite divides its transmit beams into *B* spot beams according to coverage areas, and each spot beam is equipped with $M_{s}$ antennas. The maximum transmit power of the *b*th spot beam of satellite $S_{i}$ is denoted by $p_{s_{i},b}$. Each spot beam covers *U* users, and each user is equipped with $N_{t}$ antennas. For convenience of subsequent description, the set of users served by LEO satellite $S_{i}$ is denoted as $\Phi_{s_{i}}=\left\{{\mathrm{UE}}_{s_{i},1,1},\ldots ,{\mathrm{UE}}_{s_{i},u,b},\ldots ,{\mathrm{UE}}_{s_{i},U,B}\right\}$. In this system, multiple spot beams are generated by the LEO satellites to serve ground users, while the navigation satellite provides wide-area coverage for navigation services.

### 2.2. Signal Model

Let the ICAN signal transmitted by the *b*th spot beam of LEO satellite $S_{i}$ be

(1) $$x\left(t\right)={\tilde{x}}_{s_{i},b}\cos\left(2\pi f_{s}t+\varphi_{0}^{s_{i}}\right)+x_{p}\sin\left(2\pi f_{s}t+\varphi_{0}^{s_{i}}\right),$$

where ${\tilde{x}}_{s_{i},b}$ denotes the communication signal transmitted by spot beam *b* of LEO satellite $S_{i}$ to the *U* users within its corresponding coverage area, $x_{p}$ denotes the navigation signal from the NAV satellite, $f_{s}$ denotes the forwarding frequency of the LEO satellite, and $\varphi_{0}^{s_{i}}$ denotes the initial phase of the forwarded signal from LEO satellite $S_{i}$.

Since the initial signal $x_{s_{i},u,b}$, intended for the *u*th user in spot beam *b* of LEO satellite $S_{i}$, is first precoded at the ground gateway (GW) and then transmitted to the satellite via a distortion-free feeder link before being forwarded, the communication signal ${\tilde{x}}_{s_{i},b}$ can be expressed as

(2) $$\begin{array}{c} {\tilde{x}}_{s_{i},b}=v_{s_{i},b}x_{s_{i},b}\;\\ \;\;\;\;\;=v_{s_{i},b}\sum_{u=1}^{U}\sqrt{p_{s_{i},u,b}}x_{s_{i},u,b}, \end{array}$$

where $v_{s_{i},b}\in C^{M_{s}\times 1}$ is the precoding vector for spot beam *b* of LEO satellite $S_{i}$, and $p_{s_{i},u,b}$ denotes the transmit power allocated to the *u*th user by spot beam *b* of LEO satellite $S_{i}$. Since navigation and communication signals are transmitted over two orthogonal channels, they can be separated via coherent demodulation. As a result, the navigation signal does not generate mutual interference with the communication component. Therefore, in the subsequent analysis, we focus on the communication signal, which is subject to intra-beam, inter-beam, and inter-satellite interference, while the navigation signal remains unaffected in the considered framework.

The received signal at $UE_{s_{i},u,b}$, corresponding to the communication component forwarded by the LEO satellite, can be written as

(3) $$\begin{array}{c} y_{s_{i},u,b}=H_{s_{i},u,b}v_{s_{i},b}\sum_{u=1}^{U}\sqrt{p_{s_{i},u,b}}x_{s_{i},u,b}+\sum_{l=1,l\neq b}^{B}H_{s_{i},u,l}v_{s_{i},l}x_{s_{i},l}\;\\ \;\;\;\;\;\;\;\;\;\;\;\;+\sum_{i^{\prime }=1,i^{\prime }\neq i}^{3}\sum_{b^{\prime }=1}^{B}H_{s_{i^{\prime }},u,b^{\prime }}v_{s_{i^{\prime }},b^{\prime }}x_{s_{i^{\prime }},b^{\prime }}+n_{s_{i},u,b}, \end{array}$$

where $H_{s_{i},u,l}\in C^{N_{t}\times M_{s}}$ denotes the channel matrix between spot beam l≠b of LEO satellite $S_{i}$ and $UE_{s_{i},u,b}$, $H_{s_{i^{\prime }},u,b^{\prime }}\in C^{N_{t}\times M_{s}}$ denotes the channel matrix between spot beam $b^{\prime }$ of LEO satellite $S_{i^{\prime }}$ and $UE_{s_{i},u,b}$ for $i^{\prime }\neq i$, $v_{s_{i^{\prime }},b^{\prime }}\in C^{M_{s}\times 1}$ denotes the precoding vector for spot beam $b^{\prime }$ of LEO satellite $S_{i^{\prime }}$, and $n_{s_{i},u,b}$ denotes the background noise vector.

Further, by applying the detection vector $w_{s_{i},u,b}\in C^{N_{t}\times 1}$ to the received signal $y_{s_{i},u,b}$, the processed signal can be expressed as

(4) $$\begin{array}{c} {\tilde{y}}_{s_{i},u,b}=w_{s_{i},u,b}^{H}H_{s_{i},u,b}v_{s_{i},b}\sqrt{p_{s_{i},u,b}}x_{s_{i},u,b}\;\\ \;\;\;\;\;\;\;\;\;\;\;\;+w_{s_{i},u,b}^{H}H_{s_{i},u,b}\sum_{j=1,j\neq u}^{U}v_{s_{i},b}\sqrt{p_{s_{i},j,b}}x_{s_{i},j,b}\;\\ \;\;\;\;\;\;\;\;\;\;\;\;+\sum_{l=1,l\neq b}^{B}w_{s_{i},u,b}^{H}H_{s_{i},u,l}v_{s_{i},l}x_{s_{i},l}\;\\ \;\;\;\;\;\;\;\;\;\;\;\;+\sum_{i^{\prime }=1,i^{\prime }\neq i}^{3}\sum_{b^{\prime }=1}^{B}w_{s_{i},u,b}^{H}H_{s_{i^{\prime }},u,b^{\prime }}v_{s_{i^{\prime }},b^{\prime }}x_{s_{i^{\prime }},b^{\prime }}+w_{s_{i},u,b}^{H}n_{s_{i},u,b}. \end{array}$$

In Equation (4), the first term corresponds to the desired signal, the second represents intra-beam interference from other users within the same spot beam, the third accounts for intra-satellite inter-beam interference, the fourth captures inter-satellite inter-beam interference, and the fifth denotes the noise component. Accordingly, the signal-to-interference-plus-noise ratio (SINR) of $UE_{s_{i},u,b}$ is given by

(5) $${\mathrm{SINR}}_{s_{i},u,b}=\frac{{\left|w_{s_{i},u,b}^{H}H_{s_{i},u,b}v_{s_{i},b}\right|}^{2}p_{s_{i},u,b}}{\left(\begin{array}{c} \sum_{j=1,j\neq u}^{U}{\left|w_{s_{i},u,b}^{H}H_{s_{i},u,b}v_{s_{i},b}\right|}^{2}p_{s_{i},j,b}+\sum_{l=1,l\neq b}^{B}{\left|w_{s_{i},u,b}^{H}H_{s_{i},u,l}v_{s_{i},l}\right|}^{2}p_{s_{i},l}\\ +\sum_{i^{\prime }=1,i^{\prime }\neq i}^{3}\sum_{b^{\prime }=1}^{B}{\left|w_{s_{i},u,b}^{H}H_{s_{i^{\prime }},u,b^{\prime }}v_{s_{i^{\prime }},b^{\prime }}\right|}^{2}p_{s_{i^{\prime }},b^{\prime }}+{\left|w_{s_{i},u,b}^{H}\right|}^{2}\sigma^{2}. \end{array}\right)}$$

## 3. Interference Suppression Scheme via Joint Interference Alignment and Power Allocation

In the ICAN downlink system described above, the main challenge lies in mitigating both inter-satellite and intra-satellite inter-beam interference, while also supporting multiple users per beam. To address this, we propose a joint IA and PA scheme to suppress inter-satellite and inter-beam interference, and intra-beam interference. We now detail the design of the proposed scheme in the following.

From Equation (5), it is evident that enhancing the received SINR of communication signals in the ICAN system requires an effective interference suppression scheme. Such a scheme must mitigate intra-user interference within the same spot beam and eliminate both intra-satellite inter-beam interference and inter-satellite interference among multiple LEO satellites.

To this end, the proposed scheme first applies IA to eliminate both intra-satellite inter-beam interference and inter-satellite interference. Subsequently, PA is employed to mitigate inter-user interference within each spot beam. Specifically, the proposed IA mechanism aligns both inter-satellite and intra-satellite inter-beam interference into orthogonal subspaces through a unified user detection vector, while the PA module adaptively allocates power among NOMA users within each beam. This cross-layer integration of IA and PA has not been reported in the satellite literature and is specifically tailored to the multi-satellite ICAN context.

### 3.1. Eliminating Intra-Satellite Inter-Beam and Inter-Satellite Interference

According to the 3GPP solutions for NTN, the satellite wireless interface NR-Uu enables signal forwarding in the NTN scenario, and the forwarded signal must satisfy the functionalities supported by the NTN-GW. The NTN-GW is connected to the terrestrial gNB, which, in turn, interfaces with the 5G core network via the NG interface, allowing information exchange and coordination between the access networks. Therefore, in this paper, we assume that the gateway has perfect knowledge of the channel state information (CSI) between the satellite and each user.

#### 3.1.1. Interference Elimination Based on IA

IA exploits the orthogonality between the desired signal subspace and the interference subspace to achieve interference suppression. Under the above assumptions, the intra-satellite and inter-satellite inter-beam interference in Equation (4) can therefore be completely eliminated, provided that the following conditions are satisfied.

(6) $$w_{s_{i},u,b}^{H}H_{s_{i^{\prime }},u,b^{\prime }}v_{s_{i^{\prime }},b^{\prime }}=0.$$

(7) $$w_{s_{i},u,b}^{H}H_{s_{i},u,l}v_{s_{i},l}=0.$$

(8) $$\mathrm{rank}\left(w_{s_{i},u,b}^{H}H_{s_{i},u,b}v_{s_{i},b}\right)=1.$$

Equation (6) guarantees that inter-satellite inter-beam interference is eliminated, Equation (7) ensures the elimination of intra-satellite inter-beam interference, and Equation (8) ensures that the target user within the satellite’s spot beam receives the desired signal.

Thereby satisfying the condition in Equation (6), the interference channel of the spot beam $b^{\prime }$ of inter-satellite can be aligned to the interference space $\tau_{s_{i^{\prime }},b,b^{\prime }}$ by designing the corresponding detection vector $w_{s_{i},u,b}$, i.e.,

(9) $$\mathrm{span}\left(H_{s_{i^{\prime }},1,b^{\prime }}^{H}w_{s_{i},1,b}\right)=\ldots =\mathrm{span}\left(H_{s_{i^{\prime }},U,b^{\prime }}^{H}w_{s_{i},U,b}\right)=\mathrm{span}\left(\tau_{s_{i^{\prime }},b,b^{\prime }}\right),$$

where b=1,2,…,B.

According to the principle of spatial dimension consistency, it follows that

(10) $$\left\{\begin{array}{c} H_{s_{i^{\prime }},1,b^{\prime }}^{H}w_{s_{i},1,b}=\tau_{s_{i^{\prime }},b,b^{\prime }},\;\\ H_{s_{i^{\prime }},2,b^{\prime }}^{H}w_{s_{i},2,b}=\tau_{s_{i^{\prime }},b,b^{\prime }},\;\\ \vdots \\ H_{s_{i^{\prime }},U,b^{\prime }}^{H}w_{s_{i},U,b}=\tau_{s_{i^{\prime }},b,b^{\prime }}. \end{array}\right.$$

The interference spaces of other LEO spot beams are represented by matrices, as shown in the above expression. Applying Singular Value Decomposition (SVD) yields the vectors $w_{s_{i},u,b}$ and $\tau_{s_{i^{\prime }},b,b^{\prime }}$. After aligning with the interference space $\tau_{s_{i^{\prime }},b,b^{\prime }}$, the vector $v_{s_{i^{\prime }},b^{\prime }}$ is designed such that the signal transmitted by satellite $S_{i^{\prime }}$ does not interfere with users in the target spot beam of satellite $S_{i}$, i.e., for ∀b=1,…,B, the following condition holds.

(11) $${\left[\begin{array}{ccccc} \tau_{s_{i^{\prime }},1,b^{\prime }} & \ldots & \tau_{s_{i^{\prime }},b,b^{\prime }} & \ldots & \tau_{s_{i^{\prime }},B,b^{\prime }} \end{array}\right]}^{H}v_{s_{i^{\prime }},b^{\prime }}=0$$

Similarly, according to (7), to eliminate intra-satellite inter-beam interference, $v_{s_{i},l}$ must be designed such that $v_{s_{i},l}⊥H_{s_{i},u,l}^{H}w_{s_{i},u,b}$, where l≠b. To achieve this, $v_{s_{i},l}$ is determined by the following equation.

(12) $${\left[\begin{array}{ccccc} H_{s_{i},1,1}^{H}w_{s_{i},1,b} & \ldots & H_{s_{i},u,l}^{H}w_{s_{i},u,b} & \ldots & H_{s_{i},U,B}^{H}w_{s_{i},U,b} \end{array}\right]}^{H}v_{s_{i},l}=0.$$

#### 3.1.2. Feasibility Condition Analysis

It should be noted that implementing beam interference elimination using IA requires that Equations (6) and (7) admit feasible solutions. According to Bézout’s theorem, a system of polynomial equations admits a solution only if the number of equations does not exceed the number of variables. Before conducting the feasibility analysis, we reiterate that denotes the number of receive antennas per user terminal, which provides the spatial degrees of freedom required for interference cancellation. Let the numbers of equations in (6) and (7) be $N_{e1}$ and $N_{e2}$, respectively, and the numbers of variables be $N_{v1}$ and $N_{v2}$. Therefore, the following condition holds.

(13) $$\left\{\begin{array}{c} N_{e1}=2B\sum_{t=1}^{B}U,N_{v1}=2\left(N_{t}-1\right)\sum_{t=1}^{B}U+2B\left(M_{s}-1\right),\;\\ N_{e2}=\left(B-1\right)\sum_{b=1}^{B}U,N_{v2}=\left(N_{t}-1\right)\sum_{b=1}^{B}U+B\left(M_{s}-1\right). \end{array}\right.$$

Therefore, to ensure that IA can completely eliminate the interference between spot beams (including both intra-satellite and inter-satellite), the following condition must be satisfied.

(14) $$\left\{\begin{array}{c} 2B\sum_{t=1}^{B}U\leq 2\left(N_{t}-1\right)\sum_{t=1}^{B}U+2B\left(M_{s}-1\right)\;\\ \left(B-1\right)\sum_{b=1}^{B}U\leq \left(N_{t}-1\right)\sum_{b=1}^{B}U+B\left(M_{s}-1\right) \end{array}\right.$$

To guarantee that the system admits a feasible solution, the stricter of the two inequalities in Equation (14) must be satisfied, namely, the first inequality $2B\sum_{t=1}^{B}U\leq 2\left(N_{t}-1\right)\sum_{t=1}^{B}U+2B\left(M_{s}-1\right)$. In typical multi-user scenarios, the term involving $\sum_{t=1}^{B}U$ dominates the inequality. To ensure this condition holds, a sufficient condition is derived by comparing the coefficients of the dominant terms, yielding $2B\leq 2(N_{t}-1)$. Simplifying this inequality gives, which leads directly to Equation (15).

(15) $$N_{t}\geq B+1.$$

This condition provides key insights into system scalability. From (13), IA feasibility is inherently independent of the number of users *U*, as increasing *U* does not affect the alignment condition. In contrast, feasibility explicitly depends on the number of beams *B*, since alignment requires $N_{t}\geq B+1$. Consequently, scalability with respect to *B* is attainable only if user terminals are equipped with a sufficient number of antennas.

After eliminating the inter-beam interference (including both intra-satellite and inter-satellite) in the system, the SINR of user $UE_{s_{i},u,b}$ can be expressed as

(16) $${\mathrm{SINR}}_{s_{i},u,b}=\frac{{\left|w_{s_{i},u,b}^{H}H_{s_{i},u,b}v_{s_{i},b}\right|}^{2}p_{s_{i},u,b}}{{\left|w_{s_{i},u,b}^{H}H_{s_{i},u,b}v_{s_{i},b}\right|}^{2}\sum_{j=1,j\neq u}^{U}p_{s_{i},j,b}+{\left|w_{s_{i},u,b}^{H}\right|}^{2}\sigma^{2}}.$$

### 3.2. Mitigation of Intra-Beam Inter-User Interference

In the previous subsection, the complete elimination of intra-satellite and inter-satellite inter-beam interference was achieved based on the principles of IA technology. The next step is to address intra-beam inter-user interference, i.e., the interference between different users within the same spot beam. From Equation (2), it is clear that a single spot beam serves *U* users within its coverage area, making interference between users inevitable. Therefore, this subsection focuses on interference mitigation through PA among users within the same spot beam. Before presenting the results, it is important to emphasize that the scalability of the proposed scheme is inherently non-linear. Performance is constrained by the NOMA power-sharing trade-off as *U* increases, and more critically by the IA feasibility condition $N_{t}\geq B+1$ as *B* increases, which defines the fundamental operational boundaries of the system.

#### 3.2.1. Problem Analysis

Based on the information-sharing principles of NTN and the 5G core network in 3GPP, together with the application rules of NOMA in LEO satellite communications, we assume that the users within spot beam *b* of satellite $S_{i}$ are ordered in descending channel gain. According to the principle that users with smaller channel gains are allocated higher transmission power, the allocation satisfies $p_{s_{i},1,b}\leq \ldots \leq p_{s_{i},u,b}\leq \ldots \leq p_{s_{i},U,b}$, and the power constraint for spot beam *b* is $\sum_{b=1}^{B}\sum_{u=1}^{U}p_{s_{i},u,b}\leq P$, where *P* denotes the total transmission power of satellite $S_{i}$. Users in spot beam *b* employ successive interference cancellation (SIC) for decoding, beginning with the weaker user signals, subtracting them from the received signal, and then decoding their own signal.

For simplicity, assuming perfect SIC, the SINR of user $UE_{s_{i},u,b}$ is expressed as

(17) $$\begin{array}{c} {\mathrm{SINR}}_{s_{i},u,b}=\frac{{\left|w_{s_{i},u,b}^{H}H_{s_{i},u,b}v_{s_{i},b}\right|}^{2}p_{s_{i},u,b}}{{\left|w_{s_{i},u,b}^{H}H_{s_{i},u,b}v_{s_{i},b}\right|}^{2}\sum_{j=1,j\neq u}^{U}p_{s_{i},j,b}+{\left|w_{s_{i},u,b}^{H}\right|}^{2}\sigma^{2}}\;\\ \;\;\;\;\;\;\;\;\;\;\;\;\;\;\;\;=\frac{p_{s_{i},u,b}g_{s_{i},u,b}}{\sum_{j=1}^{u-1}p_{s_{i},j,b}g_{s_{i},u,b}+1}, \end{array}$$

where $g_{s_{i,u},b}=\frac{{\left|w_{s_{i},u,b}^{H}H_{s_{i},u,b}v_{s_{i},b}\right|}^{2}}{{\left|w_{s_{i},u,b}^{H}\right|}^{2}\sigma^{2}}$ represents the normalized channel gain of user $UE_{s_{i},u,b}$. Correspondingly, the achievable rate of user $UE_{s_{i},u,b}$ is

(18) $$\begin{array}{c} R_{s_{i},u,b}=\log_{2}(1+{\mathrm{SINR}}_{s_{i},u,b})\;\\ \;\;\;\;\;=\log_{2}\left(1+\frac{p_{s_{i},u,b}g_{s_{i},u,b}}{\sum_{j=1}^{u-1}p_{s_{i},j,b}g_{s_{i},u,b}+1}\right). \end{array}$$

Based on the above analysis, intra-beam interference among users in the same spot beam can be mitigated through PA This objective is equivalent to maximizing the sum rate of users in each LEO satellite spot beam, subject to power constraints and transmission quality requirements. Accordingly, the following optimization problem is formulated.

(19) $$\begin{array}{c} \max_{{\left\{p_{s_{i},u,b}\right\}}_{u=1,b=1}^{U,B}}\sum_{b=1}^{B}\sum_{u=1}^{U}R_{s_{i},u,b}\;\\ s.t.\;C1:R_{s_{i},u,b}\geq R_{s_{i},u,b}^{th},\;u=1,\ldots ,U,\;b=1,\ldots ,B,\;\\ \;\;\;\;\;\;C2:\;0\leq p_{s_{i},1,b}\leq p_{s_{i},2,b}\leq \ldots \leq p_{s_{i},u,b},\;\;u=1,\ldots ,U,\;b=1,\ldots ,B,\;\\ \begin{array}{c} \;\;\;\;\;\;C3:\;p_{s_{i},b}\geq \sum_{u=1}^{U}p_{s_{i},u,b},\;\;b=1,\ldots ,B,\;\\ \;\;\;\;\;\;C4:\;P\geq \sum_{b=1}^{B}p_{s_{i},b},\;\;i=1,2,3. \end{array} \end{array}$$

where *P* and $p_{s_{i},b}$ represent the total transmission power of each LEO satellite and the total transmission power allocated to spot beam *b* of LEO satellite $S_{i}$, respectively. In Equation (19), constraint C1 is the QoS requirement, ensuring the minimum transmission rate for users within the spot beam. Constraint C2 specifies the power allocation among users within the spot beam according to the descending order of their channel gains. Constraints C3 and C4 impose transmission power limits for users within the spot beam and for each LEO satellite, respectively.

#### 3.2.2. Problem Transformation

Assuming no residual interference, in order to achieve more efficient SIC, the PA within the spot beam must satisfy the following conditions.

(20) $$\left\{\begin{array}{c} \left(p_{s_{i},u,b}-\sum_{j=1}^{u-1}p_{s_{i},j,b}\right)g_{s_{i},u-1,b}\geq \Delta ,\;\\ \left(p_{s_{i},u,b}-\sum_{j=1}^{u-1}p_{s_{i},j,b}\right)g_{s_{i},u-2,b}\geq \Delta ,\;\\ \vdots \\ \left(p_{s_{i},u,b}-\sum_{j=1}^{u-1}p_{s_{i},j,b}\right)g_{s_{i},1,b}\geq \Delta , \end{array}\right.$$

where Δ denotes the minimum power difference required to distinguish between two signals. This parameter is critical for the efficient operation of SIC, as it guarantees sufficient power separation between signals to prevent interference during successive decoding. Because the channel gains are ordered in descending magnitude, efficient SIC requires only the first term in Equation (20) to be satisfied, i.e.,

(21) $$\left(p_{s_{i},u,b}-\sum_{j=1}^{u-1}p_{s_{i},j,b}\right)g_{s_{i},u-1,b}\geq \Delta .$$

Moreover, constraint C1 in Equation (19) can be reformulated as

(22) $$\begin{array}{c} \log_{2}\left(1+\frac{p_{s_{i},u,b}g_{s_{i},u,b}}{\sum_{j=1}^{u-1}p_{s_{i},j,b}g_{s_{i},u,b}+1}\right)\geq R_{s_{i},u,b}^{th}\;\\ \Rightarrow p_{s_{i},u,b}\geq \sum_{j=1}^{u-1}p_{s_{i},j,b}\left(2^{R_{s_{i},u,b}^{th}}-1\right)+\frac{\left(2^{R_{s_{i},u,b}^{th}}-1\right)}{g_{s_{i},u,b}} \end{array}.$$

Based on the preceding analysis, the optimization problem in Equation (19) can be reformulated as

(23) $$\begin{array}{c} \max_{{\left\{p_{s_{i},u,b}\right\}}_{u=1,b=1}^{U,B}}\sum_{b=1}^{B}\sum_{u=1}^{U}R_{s_{i},u,b}\\ s.t.\;C1:\;p_{s_{i},u,b}\geq \sum_{j=1}^{u-1}p_{s_{i},j,b}\left(2^{R_{s_{i},u,b}^{th}}-1\right)+\frac{\left(2^{R_{s_{i},u,b}^{th}}-1\right)}{g_{s_{i},u,b}},\;\;u=2,\ldots ,U,\;b=1,\ldots ,B,\;\\ \;\;\;\;\;C2:0\leq p_{s_{i},1,b}\leq p_{s_{i},2,b}\leq \ldots \leq p_{s_{i},u,b},\;u=1,\ldots ,U,\;b=1,\ldots ,B,\;\\ \begin{array}{c} \;\;\;\;\;C3:p_{s_{i},b}\geq \sum_{u=1}^{U}p_{s_{i},u,b},\;b=1,\ldots ,B,\;\\ \;\;\;\;\;C4:P\geq \sum_{b=1}^{B}p_{s_{i},b},\;i=1,2,3,\;\\ \;\;\;\;\;C5:\left(p_{s_{i},u,b}-\sum_{j=1}^{u-1}p_{s_{i},j,b}\right)g_{s_{i},u-1,b}\geq \Delta ,\;\;u=2,\ldots ,U,\;\;b=1,\ldots ,B, \end{array} \end{array}$$

When the satellite transmission power is sufficient (a condition typically guaranteed in practice), maximizing Equation (23) is equivalent to maximizing the sum rate of users within each satellite spot beam. Consequently, the optimization problem can be further simplified as follows.

(24) $$\begin{array}{c} \max_{{\left\{p_{s_{i},u,b}\right\}}_{u=1}^{U}}\sum_{u=1}^{U}\log_{2}\left(1+\frac{p_{s_{i},u,b}g_{s_{i},u,b}}{\sum_{j=1}^{u-1}p_{s_{i},j,b}g_{s_{i},u,b}+1}\right)\;\\ s.t.C1:p_{s_{i},u,b}\geq \sum_{j=1}^{u-1}p_{s_{i},j,b}\left(2^{R_{s_{i},u,b}^{th}}-1\right)+\frac{\left(2^{R_{s_{i},u,b}^{th}}-1\right)}{g_{s_{i},u,b}},\;u=1,\ldots ,U,\;\\ \;\;\;\;\;C2:0\leq p_{s_{i},1,b}\leq p_{s_{i},2,b}\leq \ldots \leq p_{s_{i},u,b},\;u=1,\ldots ,U,\;\\ \begin{array}{c} \;\;\;\;\;C3:p_{s_{i},b}\geq \sum_{u=1}^{U}p_{s_{i},u,b},\;\\ \;\;\;\;\;C4:\left(p_{s_{i},u,b}-\sum_{j=1}^{u-1}p_{s_{i},j,b}\right)g_{s_{i},u-1,b}\geq \Delta ,\;u=2,\ldots ,U \end{array} \end{array}$$

First, constraint C5 can be reformulated as $p_{s_{i},u,b}\geq \sum_{j=1}^{u-1}p_{s_{i},j,b}+\frac{\Delta }{g_{s_{i},u-1,b}}$, for u=2,…,U. Next, constraints C1 and C2 can be further simplified.

For u=1, constraint C1 becomes

(25) $$\left\{\begin{array}{c} p_{s_{i},1,b}\geq 0\;\\ p_{s_{i},1,b}\geq \frac{\left(2^{R_{s_{i},1,b}^{th}}-1\right)}{g_{s_{i},1,b}} \end{array}\right.\Rightarrow p_{s_{i},1,m}\geq \frac{\left(2^{R_{s_{i},1,b}^{th}}-1\right)}{g_{s_{i},1,b}}.$$

For u=2,3,…,U, we obtain

(26) $$\left\{\begin{array}{c} p_{s_{i},u,b}\geq p_{s_{i},u-1,b}\;\\ p_{s_{i},u,b}\geq \sum_{j=1}^{u-1}p_{s_{i},j,b}+\frac{\Delta }{g_{s_{i},u-1,b}} \end{array}\right.\Rightarrow p_{s_{i},u,b}\geq \sum_{j=1}^{u-1}p_{s_{i},j,b}+\frac{\Delta }{g_{s_{i},u-1,b}}.$$

From the simplified form of constraint C1, it follows that constraint C2 is automatically satisfied. Therefore, the optimization problem in Equation (23) can be rewritten as follows.

(27) $$\begin{array}{c} \max_{{\left\{p_{s_{i},u,b}\right\}}_{u=1}^{U}}\sum_{u=1}^{U}\log_{2}\left(1+\frac{p_{s_{i},u,b}g_{s_{i},u,b}}{\sum_{j=1}^{u-1}p_{s_{i},j,b}g_{s_{i},u,b}+1}\right)\;\\ \begin{array}{c} s.t.\;C1:p_{s_{i},1,b}\geq \frac{\left(2^{R_{s_{i},1,b}^{th}}-1\right)}{g_{s_{i},1,b}},u=1,\;\\ \;\;\;\;\;p_{s_{i},u,b}\geq \sum_{j=1}^{u-1}p_{s_{i},j,b}\left(2^{R_{s_{i},u,b}^{th}}-1\right)+\frac{\left(2^{R_{s_{i},u,b}^{th}}-1\right)}{g_{s_{i},u,b}},u=2,\ldots ,U, \end{array}\;\\ \;\;\;C2:p_{s_{i},b}\geq \sum_{u=1}^{U}p_{s_{i},u,b},\;\\ \;\;\;C3:p_{s_{i},u,b}\geq \sum_{j=1}^{u-1}p_{s_{i},j,b}+\frac{\Delta }{g_{s_{i},u-1,b}},u=2,\ldots ,U. \end{array}$$

In the optimization problem of Equation (27), constraints C1, C2, and C3 are linear inequality constraints. According to convex optimization theory, if the objective function in Equation (27) is concave, the problem is convex. Consequently, standard convex optimization techniques can be applied to solve it.

#### 3.2.3. Optimization Problem Solving

Below, we prove that the objective function is concave through an affine transformation. Let $q_{s_{i},u,b}=\sum_{j=1}^{u}p_{s_{i},j,b}$ represent the affine transformation. Then, $p_{s_{i},u,b}=q_{s_{i},u,b}-q_{s_{i},u-1,b}$, and the objective function can be expressed as

(28) $$\begin{array}{c} \sum_{u=1}^{U-1}\log_{2}\left(\frac{q_{s_{i},u,b}g_{s_{i},u,b}+1}{q_{s_{i},u,b}g_{s_{i},u+1,b}+1}\right)+\log_{2}\left(q_{s_{i},U,b}g_{s_{i},U,b}+1\right) \end{array}$$

For u=1,2,…,U−1, let

(29) $$\log_{2}\left(\frac{q_{s_{i},u,b}g_{s_{i},u,b}+1}{q_{s_{i},u,b}g_{s_{i},u+1,b}+1}\right)=f_{s_{i},u,b}\left(q_{s_{i},u,b}\right)$$

For u=U, let

(30) $$\log_{2}\left(q_{s_{i},U,b}g_{s_{i},U,b}+1\right)=f_{s_{i},U,b}\left(q_{s_{i},U,b}\right).$$

From Equations (29) and (30), the objective function of optimization problem in Equation (27) is given by $\sum_{u=1}^{U}f_{s_{i},u,b}\left(q_{s_{i},u,b}\right)$. Further, for u=1,2,…,U−1, the first derivative of $f_{s_{i},u,b}\left(q_{s_{i},u,b}\right)$ with respect to $q_{s_{i},u,b}$ can be expressed as

(31) $$f_{s_{i},u,b}^{\prime }\left(q_{s_{i},u,b}\right)=\frac{1}{\ln2}\left(\frac{g_{s_{i},u,b}}{\left(q_{s_{i},u,b}g_{s_{i},u,b}+1\right)}-\frac{g_{s_{i},u+1,b}}{\left(q_{s_{i},u,b}g_{s_{i},u+1,b}+1\right)}\right).$$

The second derivative of $f_{s_{i},u,b}\left(q_{s_{i},u,b}\right)$ with respect to $q_{s_{i},u,b}$ is

(32) $$\begin{array}{c} {f^{\prime \prime }}_{s_{i},u,b}\left(q_{s_{i},u,b}\right)=\frac{1}{\ln2}\left(\frac{-g_{s_{i},u,b}^{2}}{{\left(q_{s_{i},u,b}g_{s_{i},u,b}+1\right)}^{2}}+\frac{g_{s_{i},u+1,b}^{2}}{{\left(q_{s_{i},u,b}g_{s_{i},u+1,b}+1\right)}^{2}}\right)\;\\ \;\;\;\;\;\;\;\;\;\;\;\;\;\;\;\;\;\;\;\;\;\;\;=\frac{1}{\ln2}\frac{\left(g_{s_{i},u+1,b}^{2}-g_{s_{i},u,b}^{2}\right)+2q_{s_{i},u,b}g_{s_{i},u,b}g_{s_{i},u+1,b}\left(g_{s_{i},u+1,b}-g_{s_{i},u,b}\right)}{{\left(q_{s_{i},u,b}g_{s_{i},u,b}+1\right)}^{2}{\left(q_{s_{i},u,b}g_{s_{i},u+1,b}+1\right)}^{2}}\;\\ \;\;\;\;\;\;\;\;\;\;\;\;\;\;\;\;\;\;\;\;\;\;\;\leq 0. \end{array}$$

For u=U, the similar results can be obtained.

(33) $$f_{s_{i},U,b}^{\prime }\left(q_{s_{i},U,b}\right)=\frac{1}{\ln2}\frac{g_{s_{i},U,b}}{\left(q_{s_{i},U,b}g_{s_{i},U,b}+1\right)}.$$

and

(34) $$f_{s_{i},U,b}^{\prime \prime }\left(q_{s_{i},U,b}\right)=\frac{1}{\ln2}\frac{-g_{s_{i},U,b}^{2}}{{\left(q_{s_{i},U,b}g_{s_{i},U,b}+1\right)}^{2}}\leq 0.$$

From the above analysis, it follows that $f_{s_{i},U,b}\;\left(q_{s_{i},U,b}\right)$ is concave. By the convexity-preserving property of affine mappings, it follows that for all u=1,…,U, the function $f_{s_{i},u,b}\;\left(q_{s_{i},u,b}\right)$ is concave, where j=1,2,…,u. Furthermore, since the objective function is composed of a finite number of independent concave functions, and the sum of concave functions remains concave, the overall objective $R_{s_{i},\mathrm{sum}}=\sum_{u=1}^{U}f_{s_{i},u,b}\;\left(q_{s_{i},u,b}\right)$ is also concave. Therefore, the optimization problem in Equation (27) is convex. Consequently, the optimization problem in Equation (27) can be solved by introducing Lagrange multipliers. Specifically, the corresponding Lagrangian function is given by

(35) $$\begin{array}{c} L\left({\left\{p_{s_{i},u,b}\right\}}_{u=1}^{U},{\left\{\lambda_{s_{i},u,b}\right\}}_{u=1}^{U},{\left\{\mu_{s_{i},u,b}\right\}}_{u=1}^{U},{\left\{\eta_{s_{i},u,b}\right\}}_{u=1}^{U}\right)\;\\ =\sum_{u=1}^{U-1}\log_{2}\left(\frac{\sum_{j=1}^{u}p_{s_{i},j,b}g_{s_{i},u,b}+1}{\sum_{j=1}^{u}p_{s_{i},j,b}g_{s_{i},u+1,b}+1}\right)+\log_{2}\left(\sum_{j=1}^{U}p_{s_{i},j,b}g_{s_{i},U,b}+1\right)\;\\ \;\;+\sum_{u=1}^{U}\lambda_{s_{i},u,b}\left(p_{s_{i},u,b}g_{s_{i}.u,b}-\left(\sum_{j=1}^{u-1}p_{s_{i},j,b}g_{s_{i},u,b}+1\right)\left(2^{R_{s_{i},u,b}^{\mathrm{th}}}-1\right)\right)\;\\ \;\;+\sum_{u=2}^{U}\eta_{s_{i},u,b}\left(p_{s_{i},u,b}g_{s_{i},u-1,b}-\sum_{j=1}^{u-1}p_{s_{i},j,b}g_{s_{i},u-1,b}-\Delta \right)\;\\ \;\;+\mu_{s_{i},b}\left(p_{s_{i},b}-\sum_{u=1}^{U}p_{s_{i},u,b}\right), \end{array}$$

where ${\left\{\lambda_{s_{i},u,b}\right\}}_{u=1}^{U}$, ${\left\{\mu_{s_{i},u,b}\right\}}_{u=1}^{U}$, ${\left\{\eta_{s_{i},u,b}\right\}}_{u=1}^{U}$ correspond to user rate satisfaction, total beam power conservation, and power separability for SIC, respectively.

The partial derivative of $L\left({\left\{p_{s_{i},u,b}\right\}}_{u=1}^{U},{\left\{\lambda_{s_{i},u,b}\right\}}_{u=1}^{U},{\left\{\mu_{s_{i},u,b}\right\}}_{u=1}^{U},{\left\{\eta_{s_{i},u,b}\right\}}_{u=1}^{U}\right)$ with respect to $p_{s_{i},u,b}$ is obtained as

(36) $$\begin{array}{c} \frac{\partial L}{\partial p_{s_{i},u,b}}=\sum_{t=u}^{U-1}\frac{g_{s_{i},t,b}-g_{s_{i},t+1,b}}{\ln2\left(q_{s_{i},t,b}g_{s_{i},t,b}+1\right)\left(q_{s_{i},t,b}g_{s_{i},t+1,b}+1\right)}+\frac{g_{s_{i},U,b}}{\ln2\left(q_{s_{i},U,b}g_{s_{i},U,b}+1\right)}\;\\ \;\;\;\;\;\;\;\;\;\;\;\;\;\;+\lambda_{s_{i},u,b}g_{s_{i},u,b}-\sum_{t=u+1}^{U}\lambda_{s_{i},t,b}g_{s_{i},t,b}\left(2^{R_{s_{i},t,b}^{th}}-1\right)-\mu_{s_{i},b}\;\\ \;\;\;\;\;\;\;\;\;\;\;\;\;\;+\eta_{s_{i},u,b}g_{s_{i},u-1,b}-\sum_{t=u+1}^{U}\eta_{s_{i},t,b}g_{s_{i},t-1,b}. \end{array}$$

According to convex optimization theory, the Karush–Kuhn–Tucker (KKT) conditions yield the optimal solution to a convex problem by jointly enforcing the optimality conditions of the objective function and the feasibility of the constraints, such that

(37) $$\left\{\begin{array}{c} \frac{\partial L}{\partial p_{s_{i},u,b}}=0,\;u=1,\ldots ,U\;\\ \lambda_{s_{i},u,b}\geq 0,\;u=1,\ldots ,U\;\\ \mu_{s_{i},b}\geq 0\;\\ \eta_{s_{i},u,b}\geq 0,\;u=2,\ldots ,U \end{array}\right.,$$

(38) $$\left\{\begin{array}{c} \lambda_{s_{i},u,b}\left(p_{s_{i},u,b}g_{s_{i},u,b}-\left(\sum_{j=1}^{u-1}p_{s_{i},j,b}g_{s_{i},u,b}+1\right)\left(2^{R_{s_{i},u,b}^{th}}-1\right)\right)=0,\;u=1,2,\ldots ,U\;\\ \mu_{s_{i},b}\left(p_{s_{i},b}-\sum_{u=1}^{U}p_{s_{i},u,b}\right)=0\;\\ \eta_{s_{i},u,b}\left(p_{s_{i},u,b}g_{s_{i},u-1,b}-\sum_{j=1}^{u-1}p_{s_{i},j,b}g_{s_{i},u-1,b}-\Delta \right)=0,\;u=2,3,\ldots ,U \end{array}\right.,$$

and

(39) $$\left\{\begin{array}{c} p_{s_{i},u,b}g_{s_{i},u,b}-\left(\sum_{j=1}^{u-1}p_{s_{i},j,b}g_{s_{i},u,b}+1\right)\left(2^{R_{s_{i},u,b}^{th}}-1\right)\geq 0,\;u=1,2,\ldots ,U\;\\ p_{s_{i},b}-\sum_{u=1}^{U}p_{s_{i},u,b}\geq 0\;\\ p_{s_{i},u,b}g_{s_{i},u-1,b}-\sum_{j=1}^{u-1}p_{s_{i},j,b}g_{s_{i},u-1,b}-\Delta \geq 0,\;u=2,3,\ldots ,U \end{array}\right..$$

In the above expressions, Equations (37)–(39) correspond to the dual feasibility, complementary slackness, and primal feasibility conditions of the optimization problem, respectively. Together, These conditions ensure that each user either fully utilizes its power constraint or remains inactive depending on whether its quality-of-service (QoS) requirement is satisfied.

For u=U, Equation (36) can be expressed as

(40) $$\frac{\partial L}{\partial p_{s_{i},U,b}}=\frac{g_{s_{i},U,b}}{\ln2\left(q_{s_{i},U,b}g_{s_{i},U,b}+1\right)}+\lambda_{s_{i},U,b}g_{s_{i},U,b}-\mu_{s_{i},b}+\eta_{s_{i},U,b}g_{s_{i},U-1,b}.$$

Setting Equation (40) equal to zero yields

(41) $$\frac{g_{s_{i},U,b}}{\ln2\left(q_{s_{i},U,b}g_{s_{i},U,b}+1\right)}=\mu_{s_{i},b}-\lambda_{s_{i},U,b}g_{s_{i},U,b}-\eta_{s_{i},U,b}g_{s_{i},U-1,b}.$$

It reflects that optimal power is achieved when the marginal gain in rate equals the marginal penalty from violating system constraints. From Equation (41), it is clear that when $\mu_{s_{i},b}=0$, the equation cannot hold. Therefore, according to Equations (3)–(36), we obtain $\mu_{s_{i},b}>0$ and $\left(p_{s_{i},b}-\sum_{u=1}^{U}p_{s_{i},u,b}\right)=0$, respectively. Consequently, the optimization problem is further reformulated as Equation (42).

(42) $$\begin{array}{c} \max_{{\left\{p_{s_{i},u,b}\right\}}_{u=1}^{U}}\sum_{u=1}^{U}\log_{2}\left(1+\frac{p_{s_{i},u,b}g_{s_{i},u,b}}{\sum_{j=1}^{u-1}p_{s_{i},j,b}g_{s_{i},u,b}+1}\right)\;\\ s.t.\;C1:p_{s_{i},u,b}\geq \max\left\{\begin{array}{c} \sum_{j=1}^{u-1}p_{s_{i},j,b}\left(2^{R_{s_{i},u,b}^{th}}-1\right)+\frac{\left(2^{R_{s_{i},u,b}^{th}}-1\right)}{g_{s_{i},u,b}},\;\\ \sum_{j=1}^{u-1}p_{s_{i},j,b}+\frac{\Delta }{g_{s_{i},u-1,b}} \end{array}\right\},\;u=2,\ldots ,U,\;\\ \;\;\;\;\;\;\;\;\;\;\;\;\;\;p_{s_{i},1,b}\geq \frac{\left(2^{R_{s_{i},1,b}^{th}}-1\right)}{g_{s_{i},1,b}},u=1,\;\\ \;\;\;\;\;\;C2:\;p_{s_{i},b}-\sum_{u=1}^{U}p_{s_{i},u,b}=0,\;u=1,\ldots ,U. \end{array}$$

Since the optimization problem involves *U* variables, at least *U* equations are required to obtain a solution. Beyond the necessary condition $p_{s_{i},b}-\sum_{u=1}^{U}p_{s_{i},u,b}=0$, U−1 additional equations are required. Since constraint C1 in this optimization problem contains two inequalities for u=2,3,…,U, both cannot hold with equality in Equation (43). Consequently, only one inequality can be active, yielding U−1 additional equations.

(43) $$\left\{\begin{array}{c} p_{s_{i},u,b}\geq \sum_{j=1}^{u-1}p_{s_{i},j,b}\left(2^{R_{s_{i},u,b}^{th}}-1\right)+\frac{\left(2^{R_{s_{i},u,b}^{th}}-1\right)}{g_{s_{i},u,b}}\;\\ p_{s_{i},u,b}\geq \sum_{j=1}^{u-1}p_{s_{i},j,b}+\frac{\Delta }{g_{s_{i},u-1,b}} \end{array}\right.$$

According to convex optimization theory, when an inequality constraint is satisfied with equality, it contributes to the optimization process, and its corresponding Lagrange multiplier is non-negative. Conversely, when an inequality constraint is not satisfied with equality, it does not affect the optimization process, and its Lagrange multiplier is zero, indicating that the constraint is redundant.

In summary, we conclude that $\mu_{s_{i},b}>0$. Furthermore, if $p_{s_{i},u,b}=\frac{2^{R_{s_{i},u,b}^{th}}-1}{g_{s_{i},u,b}}+\sum_{j=1}^{u-1}p_{s_{i},j,b}\left(2^{R_{s_{i},u,b}^{th}}-1\right)$, then we obtain

(44) $$\sum_{j=1}^{u-1}p_{s_{i},j,b}\left(2^{R_{s_{i},u,b}^{th}}-1\right)+\frac{\left(2^{R_{s_{i},u,b}^{th}}-1\right)}{g_{s_{i},u,b}}>\sum_{j=1}^{u-1}p_{s_{i},j,b}+\frac{\Delta }{g_{s_{i},u-1,b}}\Rightarrow \eta_{s_{i},u,b}=0.$$

If $p_{s_{i},u,b}=\sum_{j=1}^{u-1}p_{s_{i},j,b}+\frac{\Delta }{g_{s_{i},u-1,b}}$, we have

(45) $$\sum_{j=1}^{u-1}p_{s_{i},j,b}+\frac{\Delta }{g_{s_{i},u-1,b}}>\sum_{j=1}^{u-1}p_{s_{i},j,b}\left(2^{R_{s_{i,u},b}^{th}}-1\right)+\frac{\left(2^{R_{s_{i},u,b}^{th}}-1\right)}{g_{s_{i},u,b}}\Rightarrow \lambda_{s_{i},u,b}=0.$$

Based on the above analysis, the conclusions regarding the Lagrange multipliers are established. Furthermore, assuming that the minimum rate requirement for all users is $R_{s}^{th}$, Algorithm 1 outlines the detailed steps for solving the PA optimization problem.

### 3.3. Complexity Analysis

The computational complexity of the proposed joint IA–PA interference suppression scheme can be analyzed by separating the contributions of the two modules:

IA module: The IA stage eliminates both inter-satellite and intra-satellite inter-beam interference. Its complexity mainly arises from the singular-value-decomposition (SVD) operations used to obtain the detection and precoding vectors in Equation (10). For each of the *B* pot beams with $M_{s}$ transmit antennas and $N_{t}$ receive antennas, performing singular value decomposition (SVD) operations and solving sets of linear equations for beam precoding and user detection vectors requires complexity is approximately $O\left(2BM_{s}\left(B+U+UN_{t}\right)+\left(BM_{s}N_{t}+BM_{s}\right)\left(BU-U\right)\right)$.

PA module: PA-based intra-beam optimization, which involves convex optimization over *U* variables per beam. Using KKT conditions, the complexity order is approximately $O\left(\left(M_{s}N_{t}+M_{s}\right)BU+BU^{2}+BU\right)$. Combining both yields the overall complexity in Equation (46). Thus, the scheme scales polynomially with *B*, *U*, and $N_{t}$, and remains tractable for practical ICAN settings. Additionally, the practical runtime per cycle required is about 19.638 ms for the typical configuration in the paper.
**Algorithm 1** Optimization solution for PA1:**Input:** *B*, $p_{s_{i},b}$ for b=1,2,…,B, $R_{s}^{\mathrm{th}}$, Δ;2:**Output:** $p_{s_{i},u,b}$ for u=1,2,…,U, b=1,2,…,B3:**for** b=1 : *B* **do**4:   **for** u=U : −1 : 2 **do**5:     **if** $\frac{\Delta }{g_{s_{i},u-1,b}}+\left(p_{s_{i},b}-\sum_{j=u+1}^{U}p_{s_{i}{,}_{j},b}\right)\geq \left(p_{s_{i},b}-\sum_{j=u+1}^{U}p_{s_{i}{,}_{j},b}\right)\left(2^{R_{s}^{\mathrm{th}}}-1\right)+\frac{\left(2^{R_{s}^{\mathrm{th}}}-1\right)}{g_{s_{i}{,}_{u},b}}$ **then**6:        $\lambda_{s_{i},u,b}=0$7:        $p_{s_{i}{,}_{u},b}=\frac{\Delta }{g_{s_{i}{,}_{u-1},b}}+\left(p_{s_{i},b}-\sum_{j=u+1}^{U}p_{s_{i}{,}_{j},b}\right)$8:     **else**9:        $\eta_{s_{i},u,b}=0$10:        $p_{s_{i},u,b}=\left(p_{s_{i},b}-\sum_{j=u+1}^{U}p_{s_{i}{,}_{j},b}\right)\left(2^{R_{s}^{\mathrm{th}}}-1\right)+\frac{\left(2^{R_{s}^{\mathrm{th}}}-1\right)}{g_{s_{i}{,}_{u},b}}$11:     **end if**12:   **end for**13:   $p_{s_{i}{,}_{1},b}=p_{s_{i},b}-\sum_{j=2}^{U}p_{s_{i}{,}_{j},b}$14:**end for**

For context, benchmark schemes such as OMA and fixed PA (FP) require only linear complexity $O\left(BU\right)$, while MMF involves solving fairness-oriented optimization problems of complexity $O\left(BU^{2}\right)$. Although the proposed scheme is more complex than OMA and FP, it achieves a significantly higher sum-rate, as shown in Section 4. Compared with MMF, the proposed scheme has similar polynomial complexity but avoids the severe rate loss associated with fairness constraints. Therefore, the complexity–performance trade-off of the proposed scheme is favorable for practical ICAN deployments.

(46) $$O\left(\begin{array}{c} \left(2BM_{s}\left(B+U+UN_{t}\right)+\left(BM_{s}N_{t}+BM_{s}\right)\left(BU-U\right)\right)\;\\ +\left(\left(M_{s}N_{t}+M_{s}\right)BU+BU^{2}+BU\right) \end{array}\right).$$

## 4. Simulation Results and Analysis

This section presents simulation results evaluating the performance of the proposed interference suppression scheme in ICAN multi-user system. In accordance with the 3GPP protocol for NTN, the LEO satellite operates in the Ka-band with a downlink bandwidth of 20 GHz. Unless otherwise specified, the simulation parameters are listed in Table 1.

### 4.1. Performance Evaluation of the Proposed Interference Suppression Scheme Under Varying Parameters

**Figure 2:**
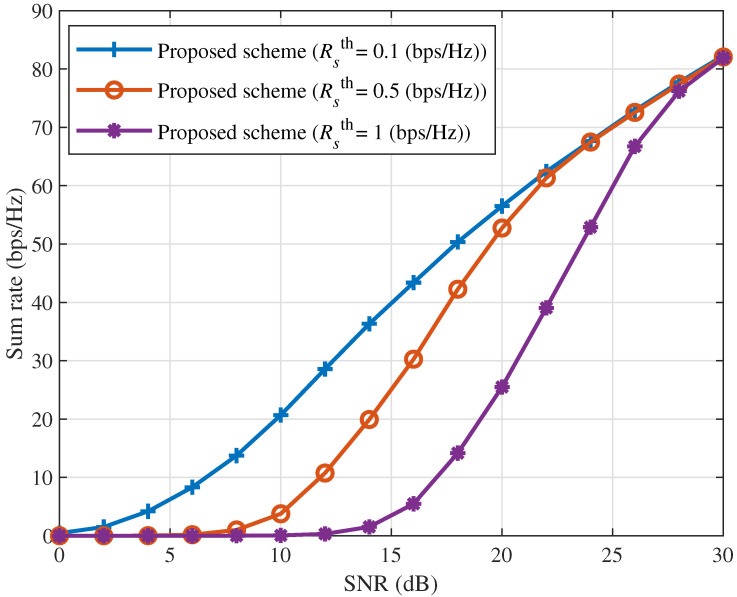
Sum rate versus SNR for different values of $R_{s}^{\mathrm{th}}$ with Δ=10 (dBm).

Figure 2 illustrates the sum rate of the ICAN system as a function of SNR for different values of $R_{s}^{\mathrm{th}}$, while Δ is fixed at 10 dBm. For a given $R_{s}^{\mathrm{th}}$, the sum rate increases monotonically with SNR. At relatively low SNRs, where Δ remains fixed, increasing $R_{s}^{\mathrm{th}}$ imposes more stringent constraints on the served users. In this regime, increasing the SNR alone may still fail to satisfy the minimum rate constraints of certain users, thereby gradually reducing the number of users that meet the QoS requirements. Consequently, as fewer users meet the QoS requirements, the overall sum rate of the ICAN system decreases. Meanwhile, the visible gap between the curves at low SNRs for different $R_{s}^{\mathrm{th}}$ values arises from insufficient transmit power. This occurs because a larger $R_{s}^{\mathrm{th}}$ imposes stricter QoS constraints, causing more users to fail the threshold, thereby reducing both the number of servable users and the total sum rate. At high SNRs (e.g., 30 dB), the transmit power becomes sufficient to satisfy all three QoS constraints. Under this condition, the number of servable users depends solely on the minimum power difference Δ, resulting in identical sum rates across all three QoS levels.

**Figure 3:**
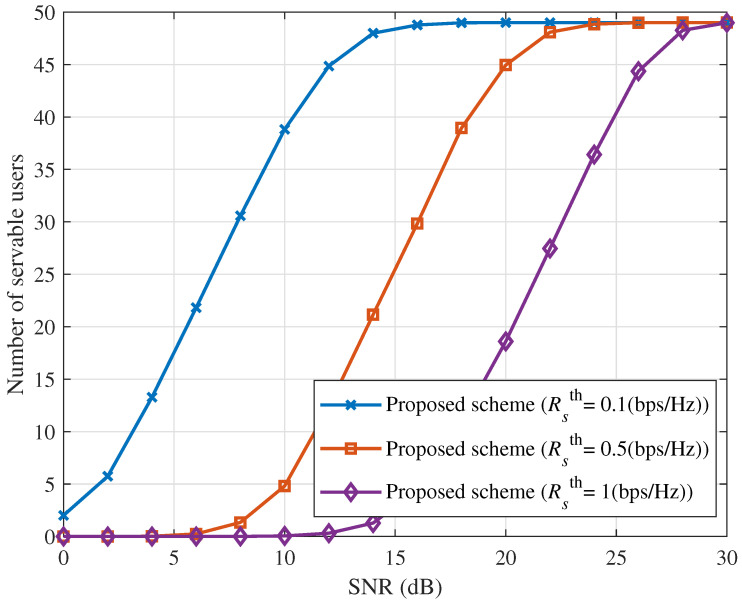
Number of servable users versus SNR for different values of $R_{s}^{th}$ with Δ=10 (dBm).

Figure 3 illustrates the number of servable users in the ICAN system versus SNR, with Δ=10dBm for different $R_{s}^{\mathrm{th}}$ values. The results indicate that the number of servable users increases with SNR. At sufficiently high SNR, the available transmit power satisfies the minimum rate requirements of all three user types. Under this condition, the number of servable users is determined solely by the minimum power difference necessary for efficient SIC. Consequently, as SNR further increases, the number of servable users under different QoS requirements converges.

**Figure 4:**
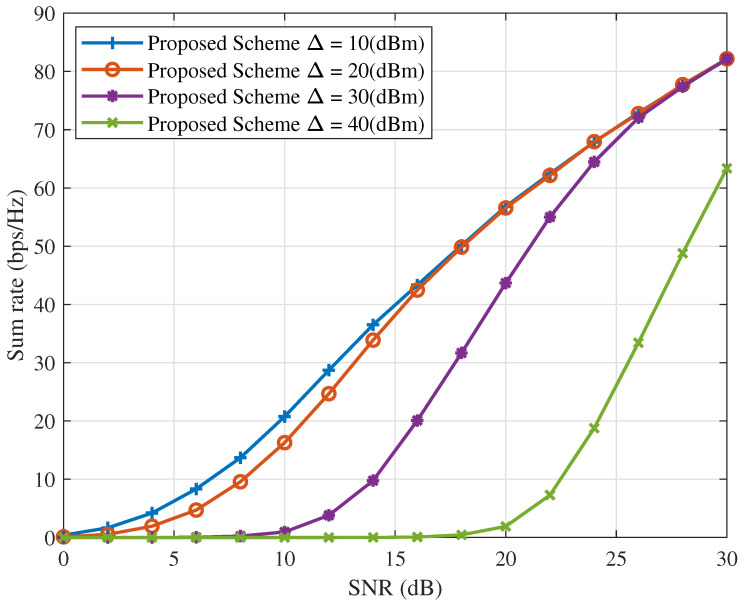
Sum rate versus SNR for different values of Δ with $R_{s}^{\mathrm{th}}=0.1\;\left(\mathrm{bps}/\mathrm{Hz}\right)$.

**Figure 5:**
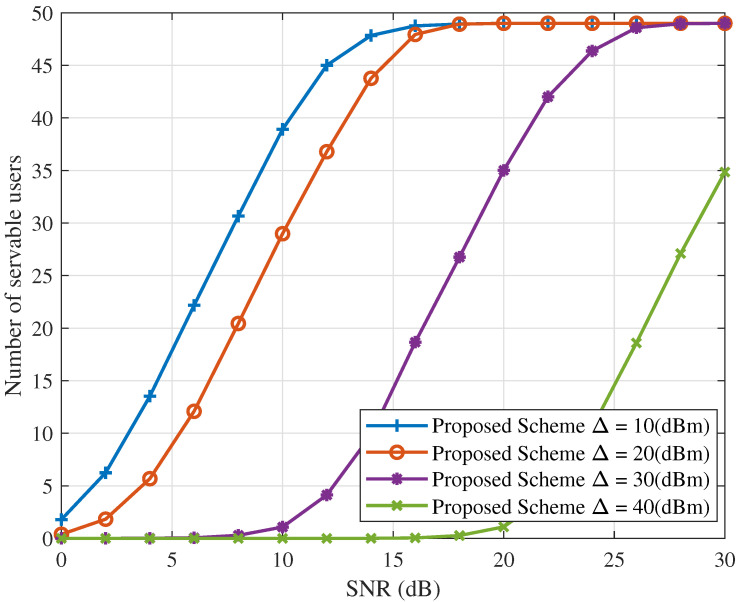
Number of servable users versus SNR for different values of Δ with $R_{s}^{\mathrm{th}}=0.1\;\left(\mathrm{bps}/\mathrm{Hz}\right)$.

Figure 4 and Figure 5 depict the ICAN system’s sum rate and number of servable users versus SNR for various values of Δ, with $R_{s}^{\mathrm{th}}$ fixed at 0.1 bps/Hz. For a given Δ, both the sum rate and the number of servable users increase with SNR under the proposed interference suppression scheme. Moreover, with $R_{s}^{\mathrm{th}}$ fixed, increasing Δ imposes more stringent conditions on the served users, reducing the number of users satisfying the QoS requirements. As fewer users satisfy the QoS constraints, the sum rate of the ICAN system decreases accordingly. These results indicate that selecting an appropriate minimum power difference Δ is critical for the proposed scheme’s performance. Therefore, Δ should be carefully determined based on the specific system requirements.

**Figure 6:**
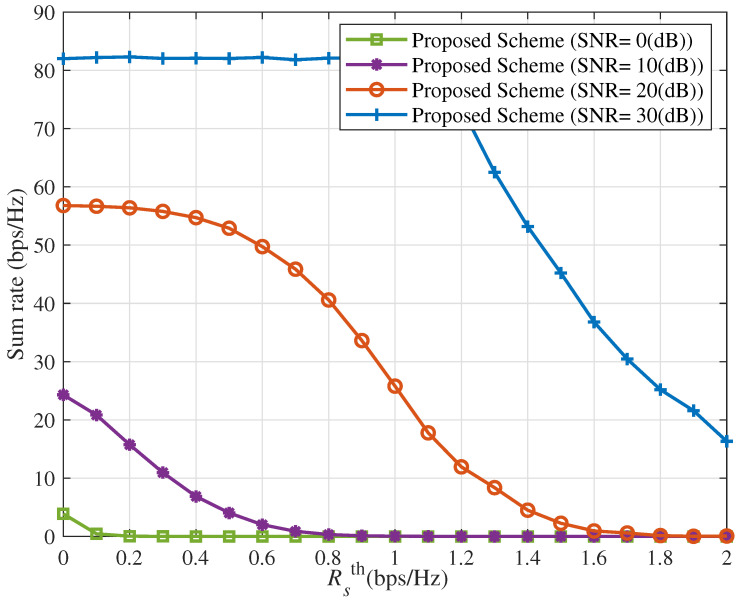
Sum rate versus versus $R_{s}^{\mathrm{th}}$ for different values of SNR with Δ=10 (dBm).

**Figure 7:**
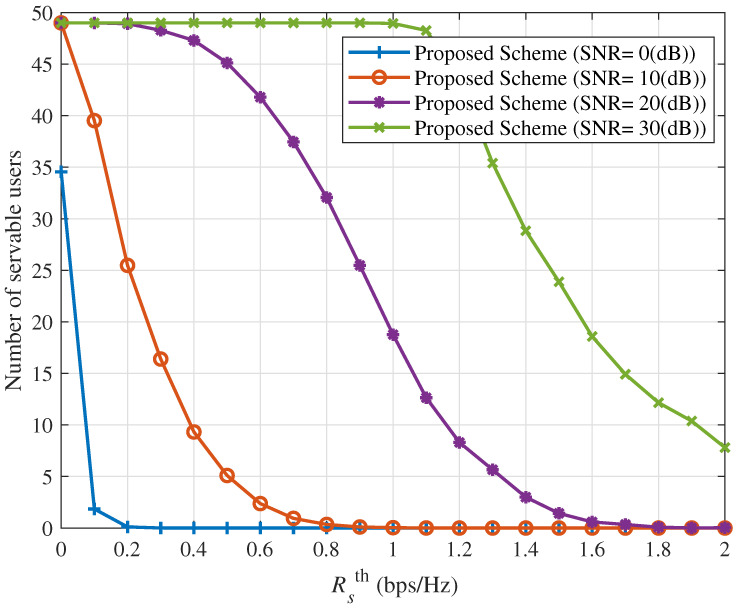
Number of servable users versus $R_{s}^{\mathrm{th}}$ for different values of SNR with Δ=10 (dBm).

Figure 6 and Figure 7 show the ICAN system’s sum rate and number of servable users versus $R_{s}^{\mathrm{th}}$ under the proposed interference suppression scheme with Δ=10 dBm for various SNR values. As $R_{s}^{\mathrm{th}}$ increases, system performance deteriorates sharply: both the sum rate and number of servable users decrease progressively, eventually approaching zero. This is because a higher $R_{s}^{\mathrm{th}}$ imposes stricter minimum rate requirements, which prevent some spot beams from serving their users, further degrading overall system performance.

**Figure 8:**
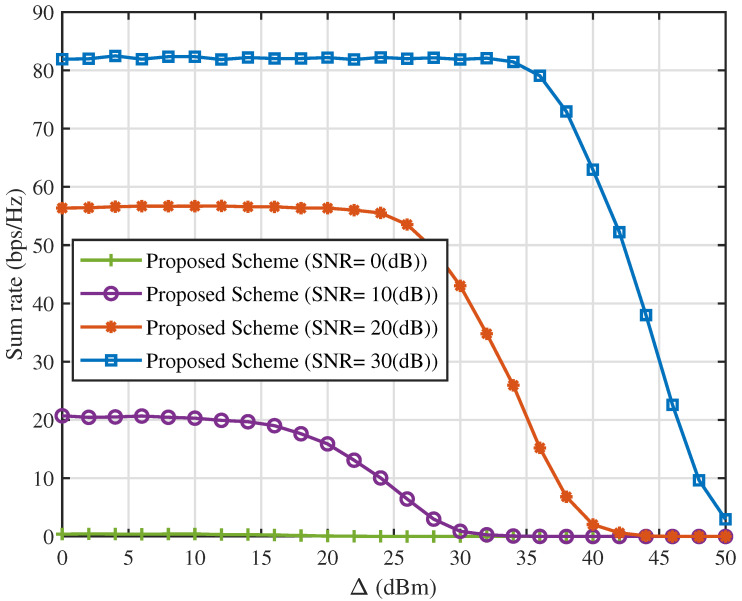
Sum rate versus Δ for different values of SNR with $R_{s}^{\mathrm{th}}=0.1\;\left(\mathrm{bps}/\mathrm{Hz}\right)$.

**Figure 9:**
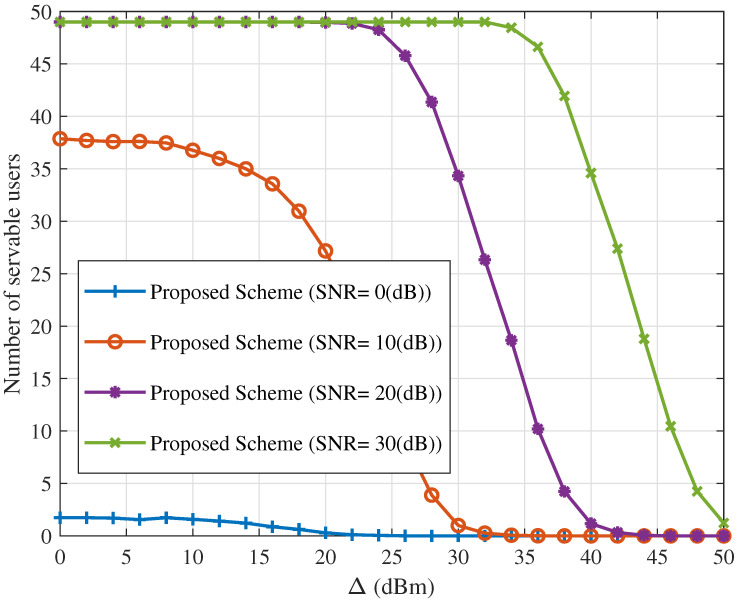
Number of servable users versus Δ for different values of SNR with $R_{s}^{\mathrm{th}}=0.1\;\left(\mathrm{bps}/\mathrm{Hz}\right)$.

Figure 8 and Figure 9 illustrate the system sum rate and number of servable users as functions of Δ for $R_{s}^{\mathrm{th}}=0.1$ bps/Hz under various SNR values using the proposed interference suppression scheme. When Δ is relatively small, users within each spot beam can easily satisfy the conditions for efficient SIC. Consequently, variations in Δ have negligible impact on both the system sum rate and the number of servable users. However, for larger values of Δ, the performance of the proposed scheme deteriorates progressively as Δ increases. This is because a larger Δ necessitates greater signal strength differences between any two users within a spot beam, imposing stricter conditions for efficient SIC. Consequently, fewer users satisfy the QoS requirements, reducing the number of served users and, in turn, decreasing the system sum rate.

### 4.2. Comparison of the Proposed Interference Suppression Scheme with Benchmark Schemes

In this subsection, we compare the per–spot-beam performance of the proposed interference suppression scheme with three representative benchmark schemes. Specifically, we selected OMA because it can completely eliminate interference by serving fewer users per spot beam, while still satisfying the minimum rate requirements of all users. The FP and MMF schemes [26] were also considered within the NOMA framework, as they manage interference through appropriate power allocation. Collectively, OMA, FP, and MMF are widely recognized and representative benchmarks, providing a fair and transparent baseline for evaluating the effectiveness of the proposed joint IA and PA scheme. Regarding the baseline schemes, OMA, FP, and MMF schemes, they are simulated under the same interference environment as the proposed scheme.

**Figure 10:**
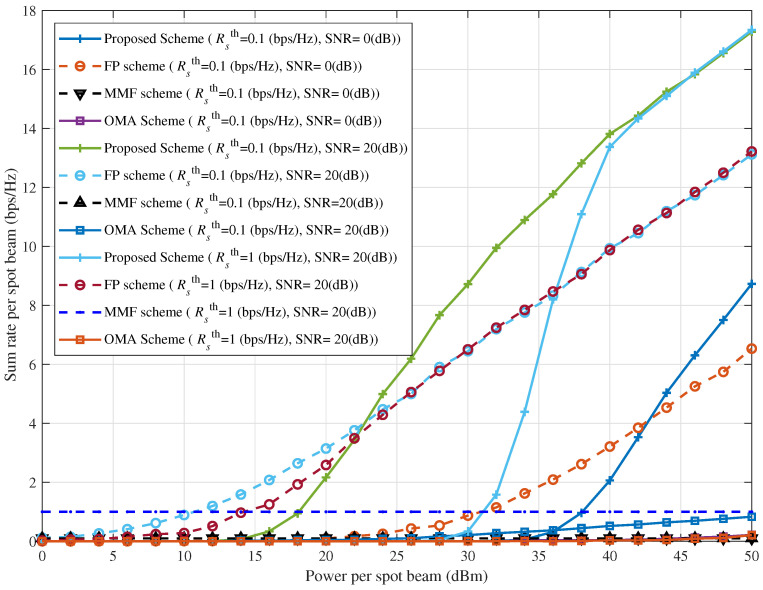
Sum rate per spot beam versus power per spot beam for different combinations SNR and $R_{s}^{\mathrm{th}}$.

Figure 10 illustrates the effect of spot beam transmit power on the sum rate for different combinations of SNR and $R_{s}^{\mathrm{th}}$ in the ICAN system, comparing the proposed interference suppression scheme with three benchmark schemes. In the FP scheme, power is allocated among users based on fixed proportional coefficients, and only users meeting the constraints are served. In contrast, the MMF scheme attempts to serve all users by relaxing the minimum rate constraint, thereby achieving absolute fairness among users sharing the same channel. However, at low transmit powers, some users’ achievable rates may fall significantly below the minimum threshold. As shown in the figure, as spot beam transmit power increases, both the proposed scheme and the FP scheme demonstrate superior sum rate performance compared with the OMA and MMF schemes. Nevertheless, because the FP scheme uses fixed proportional coefficients, it lacks flexibility and cannot surpass the proposed scheme in sum rate performance.

**Figure 11:**
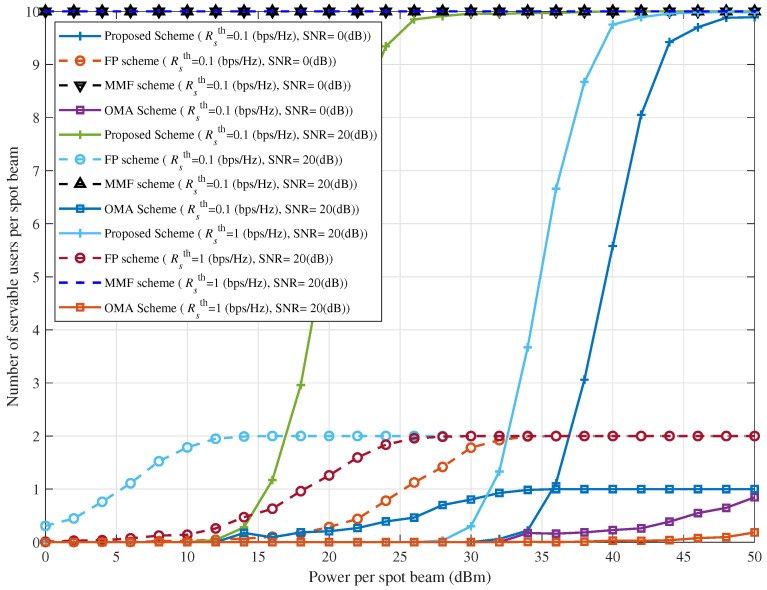
Number of servable users versus power per spot beam for different combinations of SNR and $R_{s}^{\mathrm{th}}$.

Figure 11 illustrates the effect of spot beam transmit power on the number of users that can be served within the spot beam coverage under different combinations of SNR and $R_{s}^{\mathrm{th}}$, comparing the proposed interference suppression scheme with other benchmark schemes in the integrated communication and navigation system. The MMF scheme, by relaxing the minimum rate constraint to serve all users, supports a larger number of users than the proposed and other schemes; however, its sum rate is considerably lower. The FP scheme, relying on fixed power allocation coefficients, lacks the flexibility to maximize the number of users meeting QoS requirements. The OMA scheme, which prevents intra-spot beam interference by assigning distinct time-frequency resources, serves fewer users satisfying the minimum rate constraint compared with the proposed scheme. Taken together with the results in Figure 10 and Figure 11, these observations further demonstrate the effectiveness of the proposed interference suppression scheme.

It is noted that the improved performance in Figure 2, Figure 3, Figure 4, Figure 5, Figure 6, Figure 7, Figure 8, Figure 9, Figure 10 and Figure 11 implicitly validates the IA module. Since inter-satellite and intra-satellite inter-beam interference would otherwise dominate system performance, the fact that the proposed scheme achieves markedly higher sum rates compared with benchmark schemes demonstrates that IA successfully eliminates these interference components.

## 5. Conclusions

In this paper, an interference suppression scheme based on the joint application of IA and PA is proposed to address the complex interference environment in ICAN systems. The proposed scheme first applies the IA algorithm to eliminate inter-beam interference both within and across satellites. Subsequently, intra-beam user interference is mitigated through an appropriate PA strategy, aiming to maximize the system sum rate. Simulation results demonstrate that the proposed scheme outperforms existing approaches in enhancing system performance. In summary, with the advancement of ICAN technology, the research presented in this paper offers a potential candidate solution to the challenges of complex interference and signal processing in future communication-navigation integrated systems. Future work could explore the robustness of the proposed scheme in the presence of channel estimation errors and delays, addressing how these factors can impact system performance and exploring potential mitigation techniques.

## Figures and Tables

**Table 1:** Simulation parameter settings.

Parameter Name	Value	Parameter Name	Value
Number of LEO satellites	3	Frequency band	Ka
Number of antennas of LEO satellite	64	Number of spot beam per LEO satellite *B*	6
Number of users served per spot beam *U*	10	Number of antennas per user $N_{t}$	16
Channel model	Rice fading	Rice factor *k*	3 dB

## Data Availability

Dataset available on request from the authors.

## Abbreviations

The following abbreviations are used in this manuscript:

ICAN	Integrated communication and navigation
IA	Interference alignment
PA	Power allocation
KKT	Karush–Kuhn–Tucker
GEO	Geostationary orbit
NGEO	Non-geostationary orbit
NR	New radio
LBS	Location-based services
NTN	Non-terrestrial networks
3GPP	Third Generation Partnership Project
B5G	Beyond 5G
6G	Sixth-generation
Low earth orbit	Low earth orbit
OFDM	Orthogonal frequency-division multiple
CCSK	Cyclic code shift keying
FFR	Full frequency reuse
TDM	Time-division multiplexing
MIMO	Multiple-input multiple-output
EE	Energy efficiency
ZF	Zero-forcing
NOMA	Non-orthogonal multiple access
MMF	Maximin fairness
SINR	Signal to interference plus noise ratio
CSI	Channel state information
SIC	Successive interference cancellation
SVD	Singular Value Decomposition
QoS	quality-of-service
FP	Fixed power
